# Low Temperature and Low UV Indexes Correlated with Peaks of Influenza Virus Activity in Northern Europe during 2010–2018

**DOI:** 10.3390/v11030207

**Published:** 2019-03-01

**Authors:** Aleksandr Ianevski, Eva Zusinaite, Nastassia Shtaida, Hannimari Kallio-Kokko, Miia Valkonen, Anu Kantele, Kaidi Telling, Irja Lutsar, Pille Letjuka, Natalja Metelitsa, Valentyn Oksenych, Uga Dumpis, Astra Vitkauskiene, Kestutis Stašaitis, Christina Öhrmalm, Kåre Bondeson, Anders Bergqvist, Rebecca J. Cox, Tanel Tenson, Andres Merits, Denis E. Kainov

**Affiliations:** 1 Department of Clinical and Molecular Medicine, Norwegian University of Science and Technology, 7028 Trondheim, Norway; aleksandr.ianevski@ntnu.no (A.I.); valentyn.oksenych@ntnu.no (V.O.); 2 Institute of Technology, University of Tartu, 50090 Tartu, Estonia; eva.zusinaite@ut.ee (E.Z.); nastassia.shtaida@ut.ee (N.S.); kaidi.telling@ut.ee (K.T.); tanel.tenson@ut.ee (T.T.); andres.merits@ut.ee (A.M.); 3 Department of Virology and Immunology, University of Helsinki, 00014 Helsinki, Finland; hannimari.kallio-kokko@hus.fi; 4 Helsinki University Hospital (HUS) and University of Helsinki, 00290 Helsinki, Finland; miia.valkonen@hus.fi (M.V.); anu.kantele@helsinki.fi (A.K.); 5 Institute of Medical Microbiology, University of Tartu, 50411 Tartu, Estonia; irja.lutsar@ut.ee; 6 Narva Haigla, 20104 Narva, Estonia; ellipellip@mail.ru (P.L.); nmetelitsa@gmail.com (N.M.); 7 Latvian Biomedical Research and Study Centre, 1067 Riga, Latvia; uga.dumpis@gmail.com; 8 Department of Laboratory Medicine, Lithuanian University of Health Science, 44307 Kaunas, Lithuania; astra.vitkauskiene@kaunoklinikos.lt; 9 Department of Emergency Medicine, Lithuanian University of Health Sciences, 44307 Kaunas, Lithuania; kestutis.stasaitis@kaunoklinikos.lt; 10 Department of Medical Sciences, Uppsala University, 75309 Uppsala, Sweden; christina.ohrmalm@akademiska.se (C.Ö.); kare.bondeson@akademiska.se (K.B.); anders.bergqvist@akademiska.se (A.B.); 11 Influenza Centre, Department of Clinical Science, University of Bergen, 5021 Bergen, Norway; rebecca.cox@uib.no

**Keywords:** influenza, epidemics, weather, temperature, UV

## Abstract

With the increasing pace of global warming, it is important to understand the role of meteorological factors in influenza virus (IV) epidemics. In this study, we investigated the impact of temperature, UV index, humidity, wind speed, atmospheric pressure, and precipitation on IV activity in Norway, Sweden, Finland, Estonia, Latvia and Lithuania during 2010–2018. Both correlation and machine learning analyses revealed that low temperature and UV indexes were the most predictive meteorological factors for IV epidemics in Northern Europe. Our in vitro experiments confirmed that low temperature and UV radiation preserved IV infectivity. Associations between these meteorological factors and IV activity could improve surveillance and promote development of accurate predictive models for future influenza outbreaks in the region.

## 1. Introduction

Influenza A (H1N1 and H3N2 subtypes) and B (B/Yamagata and B/Victoria lineages) viruses (IVs) cause yearly epidemics with a substantial number of infected individuals requiring primary healthcare services and hospitalization [1,2]. Up to 650,000 people are estimated to die each year from IV infections [3].

It is now becoming evident that meteorological factors are associated with seasonality of IV epidemics [4,5]. Low temperature and low humidity have been shown to enhance IV transmissibility in temperate climates at high latitudes, whereas high humidity favors outbreaks in low latitudes in tropical and subtropical zones. In mid-latitudes, semiannual outbreaks result from alternating cool and rainy conditions [6,7,8,9,10,11]. Solar or UV radiation has also been suggested to have an influence on the seasonal influenza epidemics in temperate climates [12,13]. In fact, temperature, humidity, and UV radiation among other factors (genetic and social) may be valuable indicators to include in influenza surveillance for accurate prediction of future influenza outbreaks in different climate zones.

In this study, we explored the association between six meteorological factors (temperature, UV index, humidity, wind speed, precipitation, and pressure) and IV activity in Norway, Sweden, Finland, Estonia, Latvia and Lithuania during 2010–2018. In particular, we utilized correlation analysis and machine learning modeling as well as in vitro experiments to show that low temperature and UV indexes were the most predictive meteorological factors for high IV activity. Thus, our results highlighted an important role of temperature and UV index in influenza epidemics in Northern Europe.

## 2. Materials and Methods

### 2.1. The “NorthernFlu” Consortium

To better understand the etiology of influenza epidemics in Norway, Sweden, Finland, Estonia, Latvia and Lithuania a “NorthernFlu” consortium was established in 2017. It consists of epidemiologists, clinicians and researchers from Norway, Sweden, Finland, Estonia, Latvia and Lithuania. The consortium aims to determine the impact of meteorological, societal, and genetic factors on IV stability and transmissibility and to improve prediction, prevention and treatment of severe IV infections.

### 2.2. Data Collection

Weekly statistics of the specimens’ number positive for influenza across six analyzed countries were collected from the World Health Organization (WHO) website through a FluNet global web-based tool [14]. The virological data entered into FluNet were collected from National Influenza Centers (NICs) of the Global Influenza Surveillance and Response System (GISRS) and other national influenza reference laboratories collaborating actively with GISRS, or were uploaded from the WHO regional databases. Daily statistics of five meteorological factors (temperature (°C), humidity (%), wind speed (mph), pressure (Hg), precipitation (cm)) were manually exported from the web-interface of the Weather Underground service for six capitals. The Weather Underground accumulates information from the National Weather Service (NWS), and over 250,000 personal weather stations (PWS). Time series (cloud-free erythemal) of UV index data were obtained from the operational Tropospheric Emission Monitoring Internet Service (TEMIS) ozone data archive.

### 2.3. Statistical Analysis

The FluNet database provided weekly statistics of specimens positive for influenza viruses, whereas meteorological factor data were measured daily. Therefore, we adjusted each meteorological factor (median) per week.

In order to investigate the potential relationship between meteorological factors and influenza activity (number of IV positive specimens) in all analyzed countries, correlation analysis and machine learning based modeling were applied. The Pearson correlation coefficient (r) between each meteorological factor and influenza activity was calculated using the “stats” R package with a threshold of significance, *p* = 0.05.

For the machine learning modeling, Random Forest (RF) was optimized to find the most predictive meteorological factors of influenza activity. More specifically, for each country, the time-series measurements for all six meteorological factors were used as input features (explanatory variables) and the number of IV-positive specimens as an output variable for the model. The optimal Random Forest hyper-parameters, which are estimated from the data and used to train the most accurate model, were identified using Bayesian optimization (mlrMBO R-package, version: 1.1.1), with five times repeated 10-fold cross validation. The optimized RF model (with the lowest root-mean-square error (RMSE) from cross-validation) was used to estimate the contribution of each meteorological factor as a predictive performance of IV activity. This was done by random permutation of the measurements of one of the meteorological factors, while keeping measurements for all other factors constant, followed by estimation of the impact of this procedure on model accuracy (i.e., percentage mean decrease in accuracy). RF R-package version 4.6-14 was applied for the model training, with the following hyper-parameters used for optimization: Number of trees, number of randomly sampled features at each tree split (mtry), and minimum size of terminal nodes.

To find correlation between colder winters and increases in the number of influenza-positive specimens for six countries, we applied the Cox–Stuart statistical test. In particular, the median winter temperatures (2010–2018) were calculated by taking a median of daily temperatures between 1st of December and the 28th of February. Then, the median winter temperatures were ordered by the number of total IV-positive specimens for each country each year. A Cox–Stuart statistical test was applied to the ordered median winter temperatures to find any significant increasing or decreasing trends (threshold of significance was set to *p* = 0.05).

### 2.4. Experimental Validation

Madin–Darby canine kidney (MDCK) cells were grown in Dulbecco’s Modified Eagle’s medium (DMEM; Gibco, Paisley, Scotland) supplemented with 100 U/mL penicillin and 100 μg/mL streptomycin mixture (Lonza, Cologne, Germany), 2 mM l-glutamine, 10% fetal bovine serum (FBS; Lonza, Cologne, Germany). The virus growth medium (VGM) contained 0.2% BSA, 2 mM l-glutamine, and 1 μg/mL l-1-tosylamido-2-phenylethyl chloromethyl ketone-trypsin (TPCK)-trypsin (Sigma-Aldrich, St. Louis, USA) in DMEM.

Human telomerase reverse transcriptase-immortalized retinal pigment (RPE) cells were grown in DMEM-F12 supplemented with 100 U/mL penicillin and 100 μg/mL streptomycin mixture, 2 mM l-glutamine, 10% FBS, and 0.25% sodium bicarbonate (Sigma-Aldrich, St. Louis, USA) as described previously [15]. The virus growth medium (VGM) contained 0.2% BSA, 2 mM l-glutamine, 0.35% NaHCO_3_, and 1 μg/mL TPCK-trypsin (Sigma-Aldrich) in DMEM-F12 (Gibco, Paisley, Scotland).

Human primary macrophages were derived from leukocyte-rich buffy coats from healthy blood donors. In particular, monocytes were isolated as described previously [16]. Monocytes were seeded in 96- or 6-well plates and cultured in serum free macrophage media (Gibco, Paisley, Scotland) supplemented with 10 ng/mL granulocyte macrophage colony stimulating factor (GM-CSF; Sigma-Aldrich, St. Louis, USA) and 100 U/mL penicillin and 100 μg/mL streptomycin mixture at 37 °C and 5% CO_2_ for 7 days, polarizing the monocytes into macrophages of the acute pro-inflammatory M1-phenotype. Before stimulation, the media was replaced with fresh GM-CSF free macrophage media.

GFP-expressing influenza A/PR8-NS116-GFP strain (PR8-GFP) was purified by centrifugation in sucrose gradient as previously described [17,18]. The purified virus was incubated at different temperatures for 48 h. Alternatively, the virus was exposed to UVC (λ = 254 nm) or to UVB (λ = 302 nm) using Hoefer UVC 500 Ultraviolet Crosslinker (20 J/cm^2^) or VM25/30/GX trans-illuminator as UV sources, respectively. Resulting viruses were titered on MDCK cells using plaque assay as described previously [15]. The virus titers were determined by calculating the plaque forming units (PFU) for each sample and were expressed as PFU/mL.

RPE cells were infected with the viruses at a multiplicity of infection (moi) of 1. After 24 h GFP expression was measured in infected cells using a fluorescent microscope (Zeiss Observer Z1, Zaventem, Belgium). Viability of cells were measured using Cell Titer Glow assay (Promega, Madison, USA). The luminescence was read with a PHERAstar FS plate reader (BMG Labtech, Ortenberg, Germany).

The A/Helsinki/P18/2009(pdm09) virus was isolated from nasopharyngeal aspirates of Finnish patients in 2009 [19]. The viral genome was sequenced (GeneBank accession numbers: JQ173161–JQ173168). The virus was exposed to different temperatures, UVB or UVC radiation. Resulting viruses were titered on MDCK cells using plaque assay as described previously [15]. The virus titers were determined by calculating the plaque forming units (PFU) for each sample and expressed as PFU/mL.

Macrophages were infected with mock, temperature/UV-treated or untreated viruses for 36 h. The viability of cells were measured using Cell Titer Glow assay using a PHERAstar FS plate reader.

## 3. Results

### 3.1. Influenza Virus Activity during 2017–2018 Season

**Figure 1:**
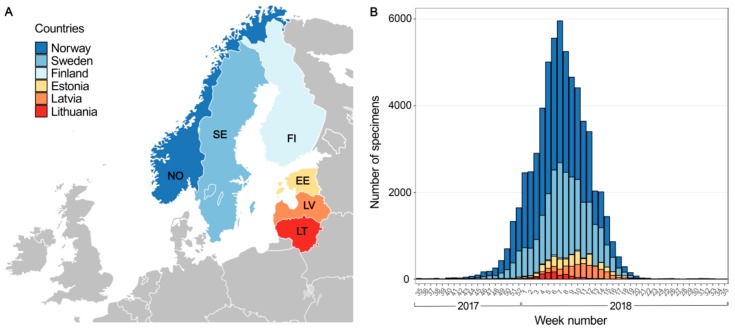
Weekly number of detections of influenza viruses in six Northern European countries, 2017–2018. (**A**). Map showing six Northern European countries included in the analysis. A blank map of Europe in SVG format from Wikimedia Commons, was used as a template. (**B**) Stacked bar chart representing the number of influenza-positive specimens distributed across six countries between week 35 of 2017 and week 34 of 2018. Each country is shown as a bar in the same color as (A).

A total of 62,296 IV-positive specimens were collected from Norway, Sweden, Finland, Estonia, Latvia and Lithuania from 1 September 2017 to 31 August 2018 (Figure 1). The highest number of IV-positive specimens was collected from Norway (34,895), whereas the lowest number of samples was from Finland (231). Among positive specimens in six countries, only 11% were classified: 3744 were influenza A viruses (71% of A/H3 and 29% of A/H1) and 2866 were influenza B viruses (97% of B/Yamagata and 3% of B/Victoria). Most IVs were detected between the 1st and 14th weeks of 2018 (Figure 1), while the peak of influenza occurred between 29 January, 2018 and 11 March, 2018 (5th to 10th weeks). The only exception was Latvia, where the peak of influenza activity occurred between the 9th and 12th weeks (Figure S1).

### 3.2. Temperature and UV Index Are the most Predictive Meteorological Factors of IV Epidemic in 2017–2018 in Northern Europe

We obtained daily statistics for six meteorological factors (temperature, UV index, humidity, wind speed, atmospheric pressure, and precipitation) from the web-interface of Weather Underground and operational Tropospheric Emission Monitoring Internet services for Norway, Sweden, Finland, Estonia, Latvia and Lithuania from 1 September 2017 to 31 August 2018 (Figures S1 and S2).

We analyzed correlation between each meteorological factor and IV activity to find significant associations and to measure the strength of linear association between variables using the Pearson correlation coefficient. As the correlation analysis does not always provide an accurate picture of cause and effect, especially in nonlinear systems, where interdependence between variables is complex, we validated our results by optimizing a machine learning Random Forest model. The model was optimized for each of the analyzed countries with an average explained variation of 71%.

**Figure 2:**
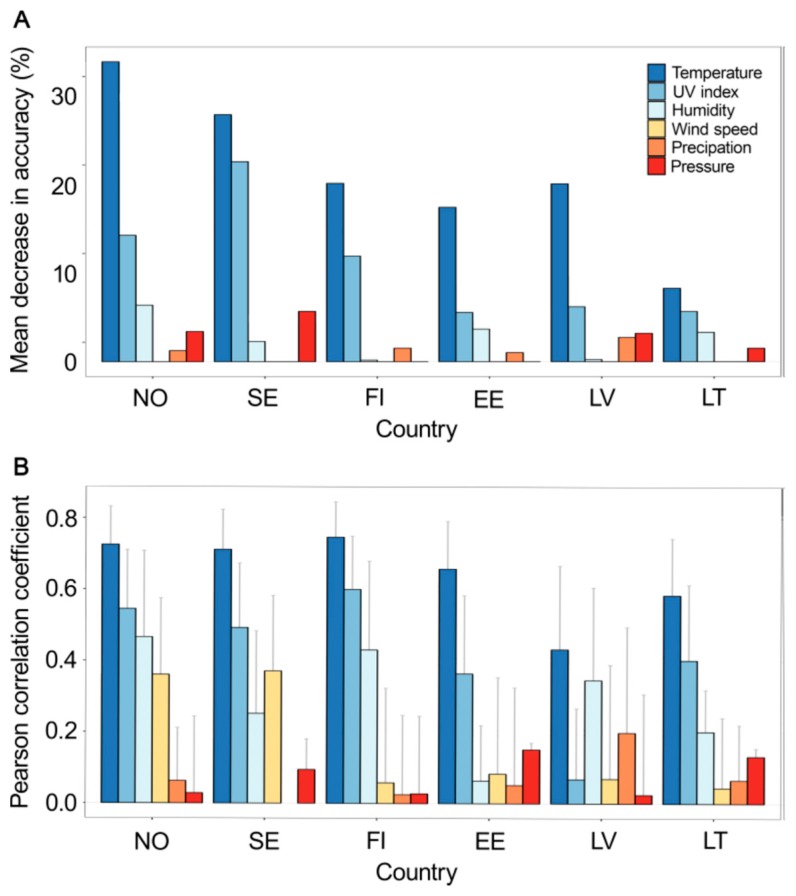
Association between six meteorological factors (temperature, UV index, humidity, wind speed, precipitation, and pressure) and influenza activity in six Northern Europe countries (Norway, Sweden, Finland, Estonia, Latvia and Lithuania) between week 35 of 2017 and week 34 of 2018. (**A**) Contribution of each meteorological factor to predictive performance of a machine learning Random Forest model trained to predict influenza activity. The contribution is measured as percentage mean decrease in accuracy (the higher the bar, the more important the factor). (**B**) Correlation between meteorological factors and influenza activity. Absolute values of Pearson correlation coefficient (95% confidence interval) are shown.

Both correlation and machine learning analyses revealed that temperature was the most predictive meteorological factor of influenza activity in all six Northern European countries (Figure 2). UV index was the second most predictive in all countries, except Latvia. The humidity was the third most important factor associated with IV activity. Other meteorological factors, including wind speed, precipitation, and pressure showed low correlation with the peaks of IV activity and were not among the most predictive features of the machine learning Random Forest model.

### 3.3. Temperature and UV Index Are the Most Predictive Meteorological Factors of IV Epidemics in 2010–2017 in Northern Europe

**Figure 3:**
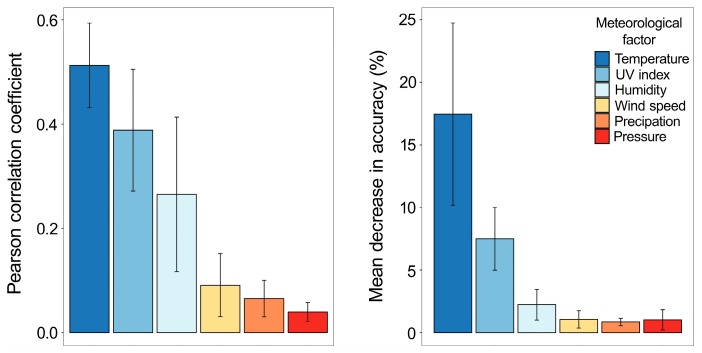
Association between meteorological factors (temperature, UV index, humidity, wind speed, precipitation, and pressure) and influenza activity in six Northern Europe countries averaged for the period from week 35 of 2010 and week 34 of 2017. (**Left**) Correlation analysis between meteorological factors and influenza activity. (**Right**) Contribution of each meteorological factor to predictive performance of machine-learning Random Forest models trained to predict the influenza virus (IV) activity in six Northern European countries. Contribution is measured as percentage mean decrease in accuracy (the higher the bar, the more important the feature is). Both metrics are averaged for the period from 2010 to 2017 epidemic seasons. Each error bar shows a 95% confidence interval for the mean of six countries (*n* = 6).

To confirm our finding, we analyzed associations between six meteorological factors and IV activity in six European countries during 2010–2017. Both low temperature and low UV index remained the most predictive meteorological factors of IV activity peaks (Figure 3). Interestingly, the peaks of IV activity occurred on average at −1.9 °C (range −19.9 °C to 26.0 °C) and with a UV index of 0.7 (range 0.13 to 7.1). However, colder winters contributed to an increase of IV activity only in Finland and Lithuania (Figure S3).

### 3.4. Low Temperature and Low UV Radiation Preserve IV Infectivity In Vitro

To validate the association between IV activity and low temperature and UV indexes, we performed in vitro experiments. Purified GFP-encoding influenza A/Puerto Rico/1934 virus (PR8-GFP) was incubated at different temperatures for 48 h or exposed to UVC or UVB radiation for different times. The resulting virus preparations were subjected to titration on MDCK cells. Exposure to high temperatures or prolonged UVC, but not UVB radiation, lowered virus infectivity (Figure S4A,B)

**Figure 4:**
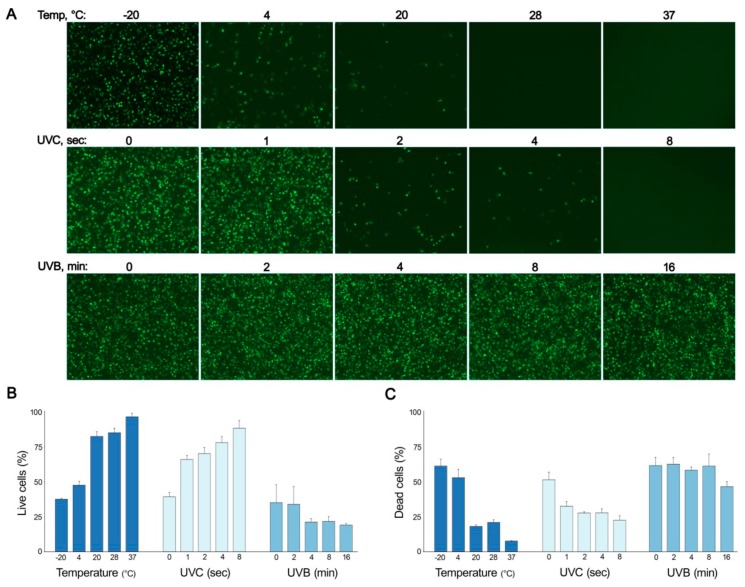
Effect of temperature and UV radiation on infectivity of GFP-encoding influenza A virus (PR8-GFP). (**A**) PR8-GFP was incubated at indicated temperatures for 48 h or exposed to UVB or UVC radiation for the indicated times. Human telomerase reverse transcriptase-immortalized retinal pigment (RPE) cells were subsequently infected with the virus. GFP expression was visualized using fluorescent microscopy. The size of each image corresponds to 1000 × 1250 μM^2^. (**B**) Viruses were obtained and RPE cells were infected as for (A). Viability of cells were measured using Cell Titer Glow assay. Mean ± SD, *n* = 3. (**C**) Viruses were obtained and RPE cells were infected as for panel A. Death of cells were measured using Cell Tox Green assay. Mean ± SD, *n* = 3.

Next, we infected human RPE cells with temperature-, UVB- or UVC-exposed viruses and monitored virus-mediated GFP expression. We observed that increasing temperature and prolonged exposure to UVC, but not UVB, reduced A/H1N1-mediated GFP expression in infected cells (Figure 4A). Moreover, viruses exposed to high temperature or prolonged UVC radiation were unable to kill RPE cells (Figure 4B,C).

We performed similar experiment using clinical A/H1N1(pdm09) influenza isolate and human PBMC-derived macrophages. Viruses exposed to high temperature or UVC radiation were neither viable nor able to kill macrophages, consistent with above results (Figure S4C,D). Altogether, these results suggest that low temperature and UV radiation preserved IV infectivity.

## 4. Discussion

Previous studies have shown that absolute humidity and temperature are major determinants of IV activity in temperate climates [7,20]. In this study, we used correlation and machine learning analyses to quantify the impact of six meteorological factors (temperature, UV index, humidity, wind speed, atmospheric pressure, and precipitation) on the IV activity in six Northern European countries (Norway, Sweden, Finland, Estonia, Latvia and Lithuania) during 2010–2018. We demonstrated that low temperature, UV indexes and humidity were associated with annual peaks of IV activity. Humidity was a less important predictor of IV activity compared to temperature and UV index. These results could be due to a statistical anomaly (UV and humidity were very close in their indices of association) or a specific phenomenon caused by the long and dark winters of Northern Europe.

Our experiments with UVB-, UVC- or temperature-exposed viruses showed that low temperature and UVB preserved infectivity of IV in vitro. It should be noted, that exposure to UVC radiation inactivated the virus. However, UVC, by contrast to UVB, is absorbed by atmosphere and, therefore, could not reach the earth surface and affect IV-host interaction.

Previous association studies showed that warmer winters could contribute to the decrease in IV activity in Europe and the US [21,22]. In particular, mild epidemics were associated with warm winters, which were followed by cold winters with severe IV outbreaks with early onset. It was proposed that fewer people were infected with influenza during warm winters, thereby leaving an unnaturally large fraction of susceptible individuals in the population going into the next season leading to early and severe epidemics. Our study does not support an association between warmer winters and a decrease in IV activity, because such a trend was observed only for two of six analyzed countries for the period from 2010 to 2018. Hence, there must be other factors such as host age and immune competence that influence IV activity, which are outside of the scope of this study.

It was shown that low winter temperatures correlated with both influenza incidence and global mortality, which mainly affected those aged 65 and older [21,23,24]. Low UV indexes could also be associated with global mortality. Indeed, data from six Northern European countries reported to the EuroMOMO project showed an excess mortality from all causes between the beginning of Januaries and the end of Februaries in 2010–2018, and coincided with IV epidemic peaks, low temperature and low UV indexes.

## 5. Conclusions

Inclusion of temperature, UV index and other meteorological parameters in IV surveillance systems could further our understanding of virus stability and transmissibility in the world, and help to develop accurate predictive models of influenza epidemics. Moreover, the combination of epidemiological, meteorological and genetic studies could unravel the evolution of influenza viruses and, consequently, improve early intervention and long-term control strategies of future influenza outbreaks.

## Supplementary Materials

The following are available online at https://www.mdpi.com/1999-4915/11/3/207/s1. Figure S1: Weekly influenza virus activity and daily temperatures across six Northern European countries between 1 September 2017 and 31 August 2018. Figure S2: Weekly influenza virus activity and daily UV indices across six Northern European countries between 1 September 2017 and 31 August 2018. Figure S3: Graph showing relation between the number of influenza-positive specimens and median winter temperatures (2010–2018) in six Northern European countries. Figure S4: Effect of temperature and UV radiation on infectivity of influenza A/Puerto Rico/8/1934 (PR8-GFP) and A/Helsinki/P18/2009(pdm09) strains.

## Abbreviations

IV	influenza virus
IAV	influenza A virus
UV	ultraviolet
GFP	green fluorescent protein
RPE	human telomerase reverse transcriptase-immortalized retinal pigment cells
moi	multiplicity of infection
hpi	hours post infection
WHO	World Health Organization
NIC	National Influenza Center
GISRS	Global Influenza Surveillance and Response System
NWS	National Weather Service
PWS	personal weather stations
TEMIS	Tropospheric Emission Monitoring Internet Service
RF	Random Forest
RMSE	root-mean-square deviation
DMEM	Dulbecco’s Modified Eagle’s medium
TPCK-trypsin	l-1-tosylamido-2-phenylethyl chloromethyl ketone-trypsin
