# Supplementary material for: Low Temperature and Low UV Indexes Correlated with Peaks of Influenza Virus Activity in Northern Europe during 2010–2018
Source: Viruses. 2019 Mar 1;11(3):207. doi: 10.3390/v11030207 (PMC6466003; doi:10.3390/v11030207)
Supplement: Supplementary file 1 [file viruses-11-00207-s001.pdf]

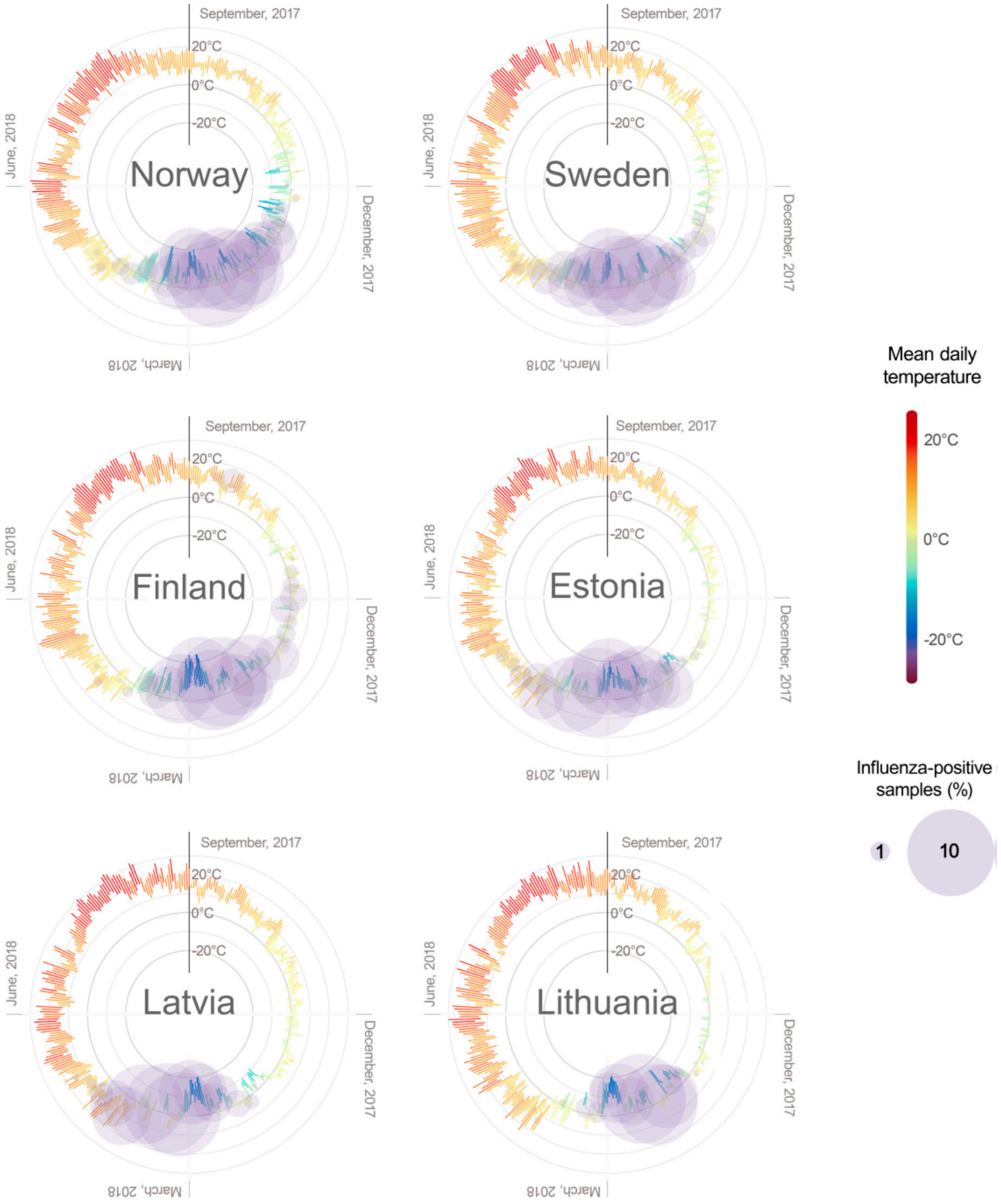

Figure S1. Weekly influenza virus activity and daily temperatures across six Northern European countries between 1 September, 2017 and 31 August, 2018. Six circular weather graphs show the daily temperature between 1 September, 2017 and 31 August, 2018 in Norway, Sweden, Finland, Estonia, Latvia and Lithuania. Each bar on a circular graph represents the maximum and minimum daily temperature. Circles show the percentage of total number of registered IV-positive samples for each country during indicated period.

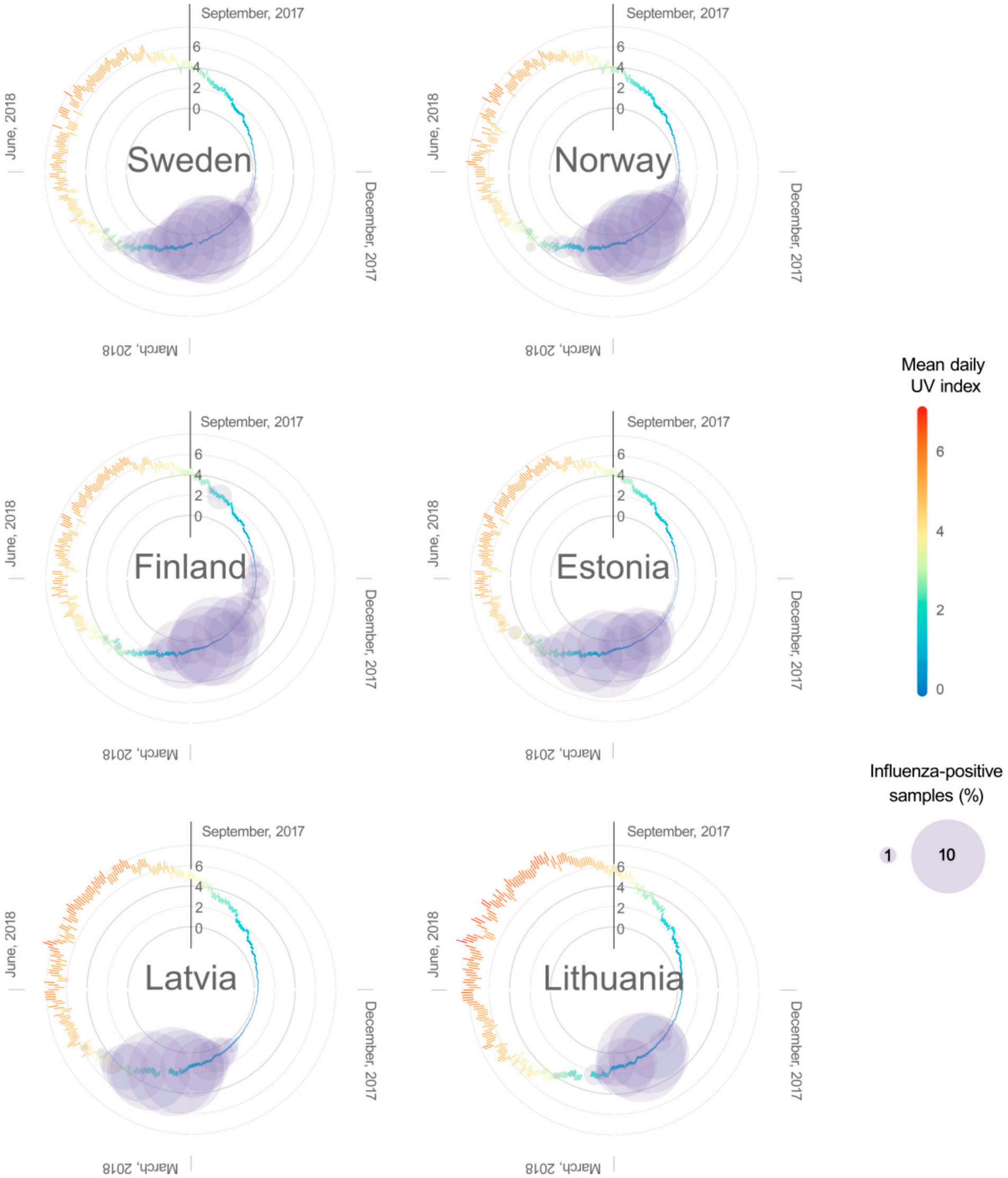

Figure S2. Weekly influenza virus activity and daily UV indices across six Northern European countries between 1 September, 2017 and 31 August, 2018. Six circular weather graphs show the daily UV index between 1 September, 2017 and 31 August, 2018 in Norway, Sweden, Finland, Estonia, Latvia and Lithuania. Each bar on a circular graph represents the maximum and minimum daily UV index. Purple-grey circles show the percentage of total number of registered IV-positive samples for each country during the indicated period.

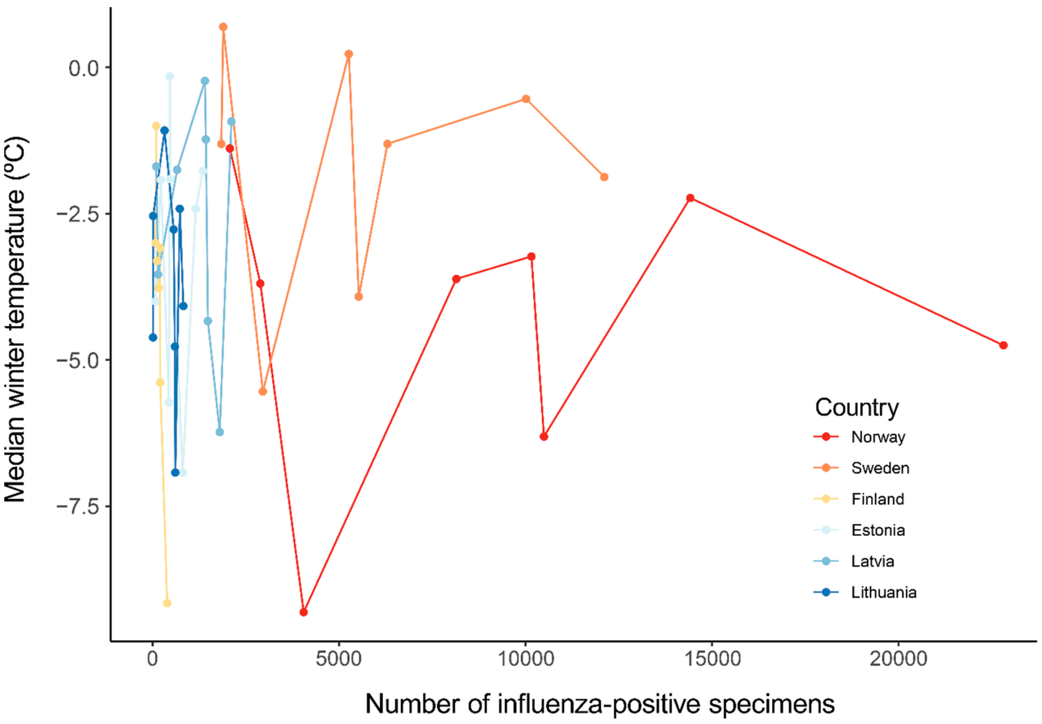

Figure S3. Graph showing relation between the number of influenza-positive specimens and the median winter temperatures (2010–2018) in six Northern European countries. The Cox–Stuart statistical test was used to check whether the data has increasing/decreasing trends (p-values: Lithuania, 0.02; Estonia, 0.50; Finland, 0.02; Latvia, 0.16; Sweden, 0.69; Norway, 0.31).

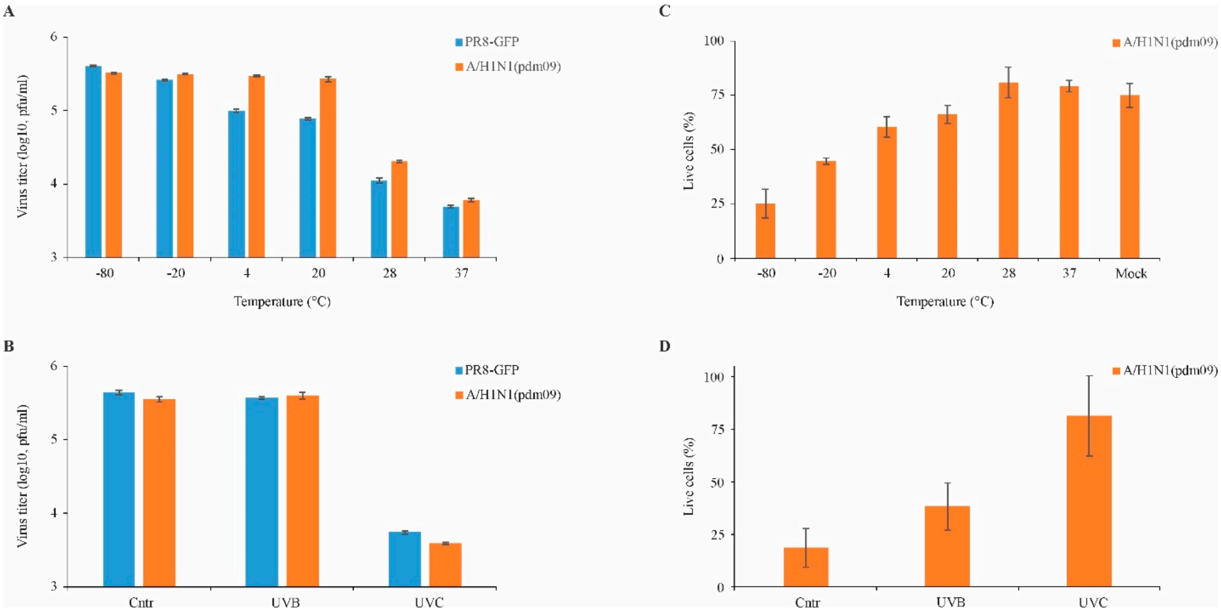

Figure S4. Effect of temperature and UV radiation on infectivity of influenza A/Puerto Rico/8/1934 (PR8-GFP) and A/Helsinki/P18/2009(pdm09) viruses. (**A**,**B**) The viruses were incubated at indicated temperatures for 48 h or exposed to UVB or UVC radiation for 16 min or 8 s, respectively. Resulting viruses were titered on Madin–Darby canine kidney (MDCK) cells using plaque assay. The virus titers were determined by calculating the plaque forming units (PFU) for each sample and expressed as PFU/ml. Mean ± SD, n=3. (**C**,**D**) Viruses were obtained as for (A). Human PBMC-derived macrophages were infected with the viruses. Viability of cells were measured using Cell Titer Glow assay. Mean ± SD, n=3.
